# Supplementary material for: Targeted Mutations Produce Divergent Characteristics in Pedigreed Sake Yeast Strains
Source: Microorganisms. 2023 May 12;11(5):1274. doi: 10.3390/microorganisms11051274 (PMC10222758; doi:10.3390/microorganisms11051274)
Supplement: Supplementary file 1 [file microorganisms-11-01274-s001.zip › GE Figure_Sup_230511_ver2_nora.pdf]

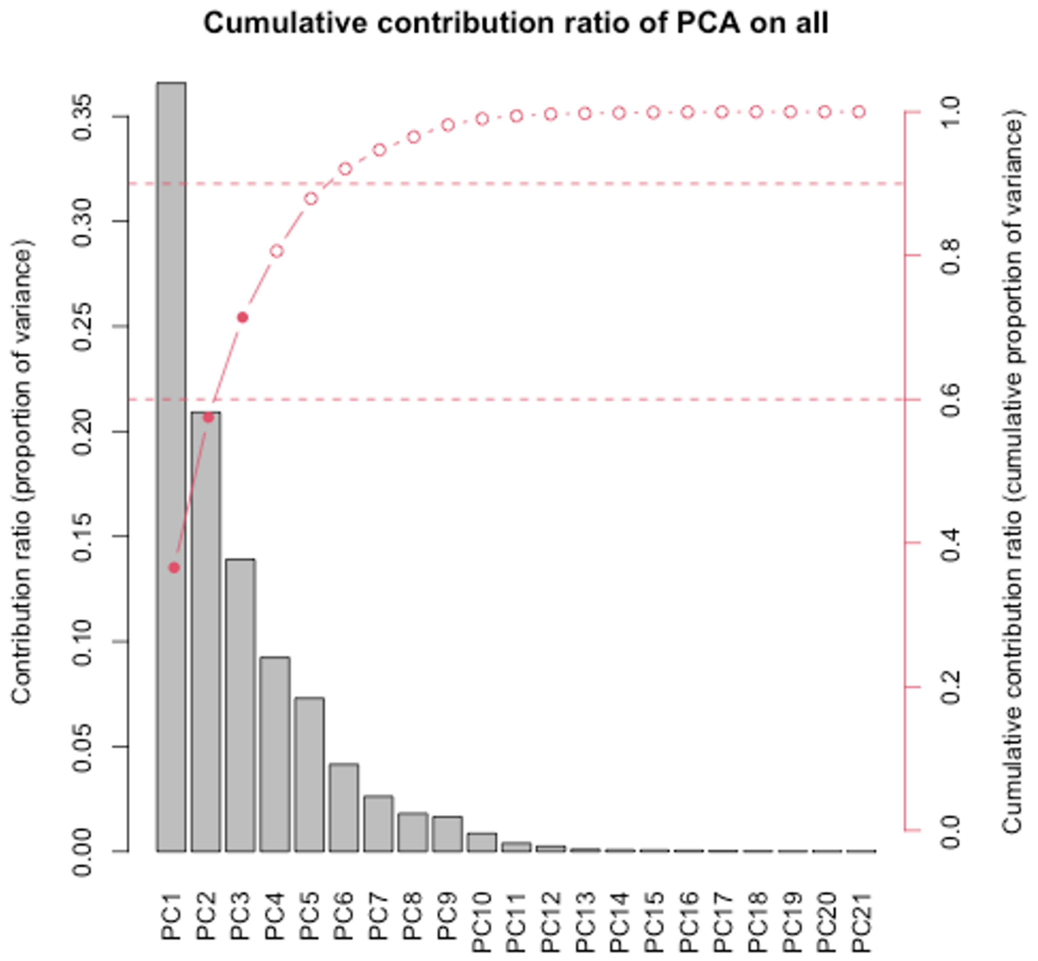

Figure S1. Cumulative contributions of principal components after PCA with 21 components.

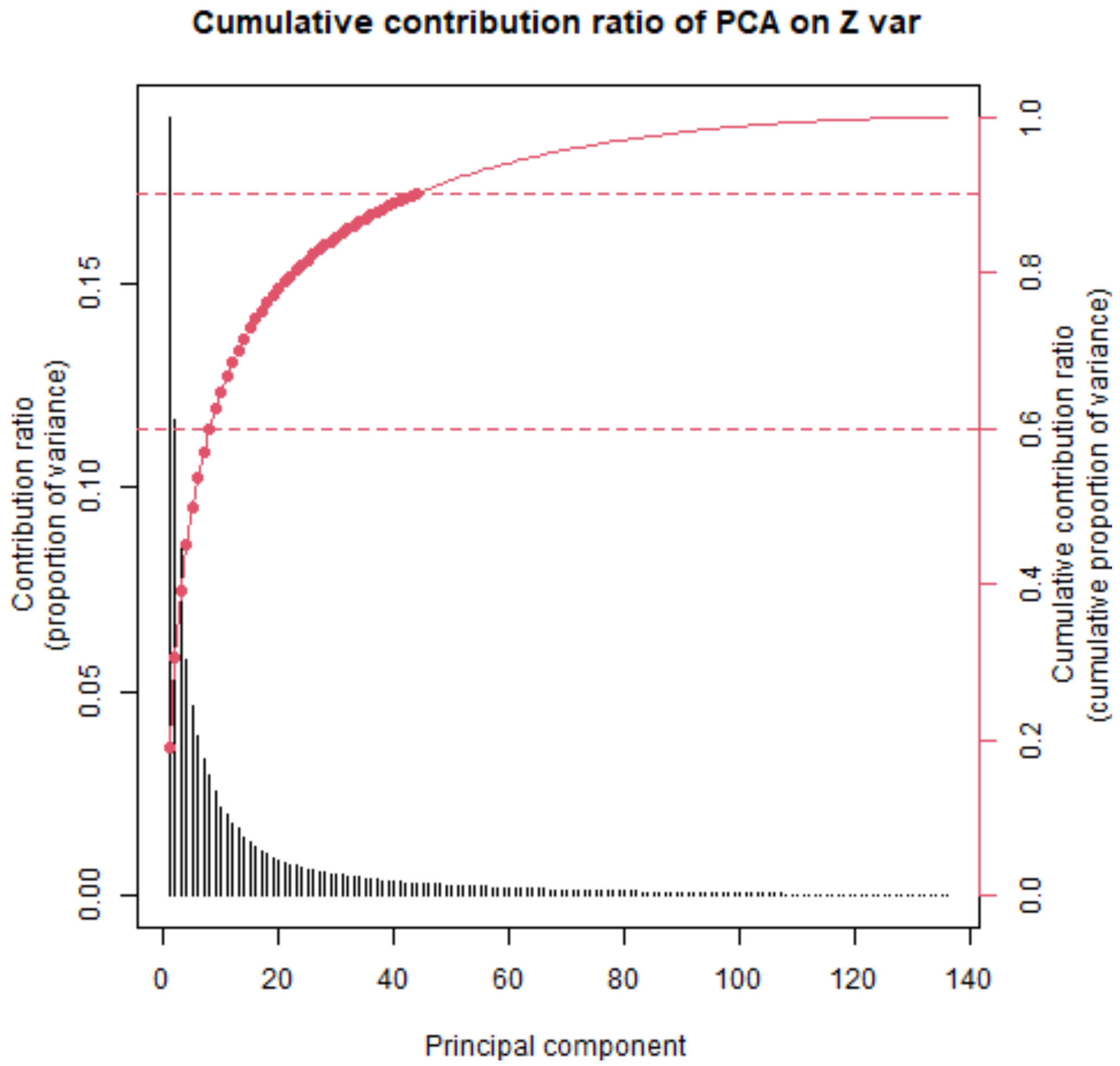

Figure S2. Cumulative contributions of principal components after PCA with a variance–covariance matrix to the Z-values of each sample in all strains for all 501 traits. From the PCA of all samples, the cumulative contribution ratios of the first 44 principal components reached 90%.

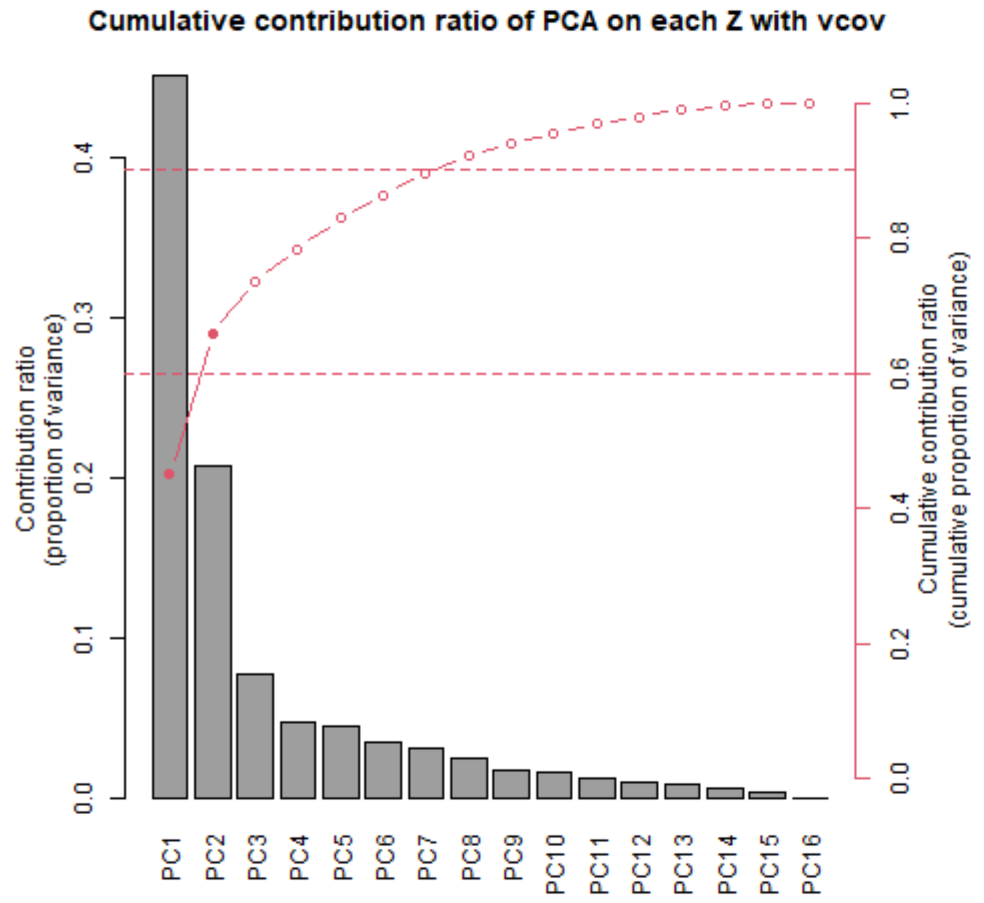

Figure S3. Cumulative contributions of principal components after PCA with a variance–covariance matrix to the Z-values of all 16 genome-edited strains. From the PCA of all strains, the cumulative contribution ratios of the first six principal components reached 90%.

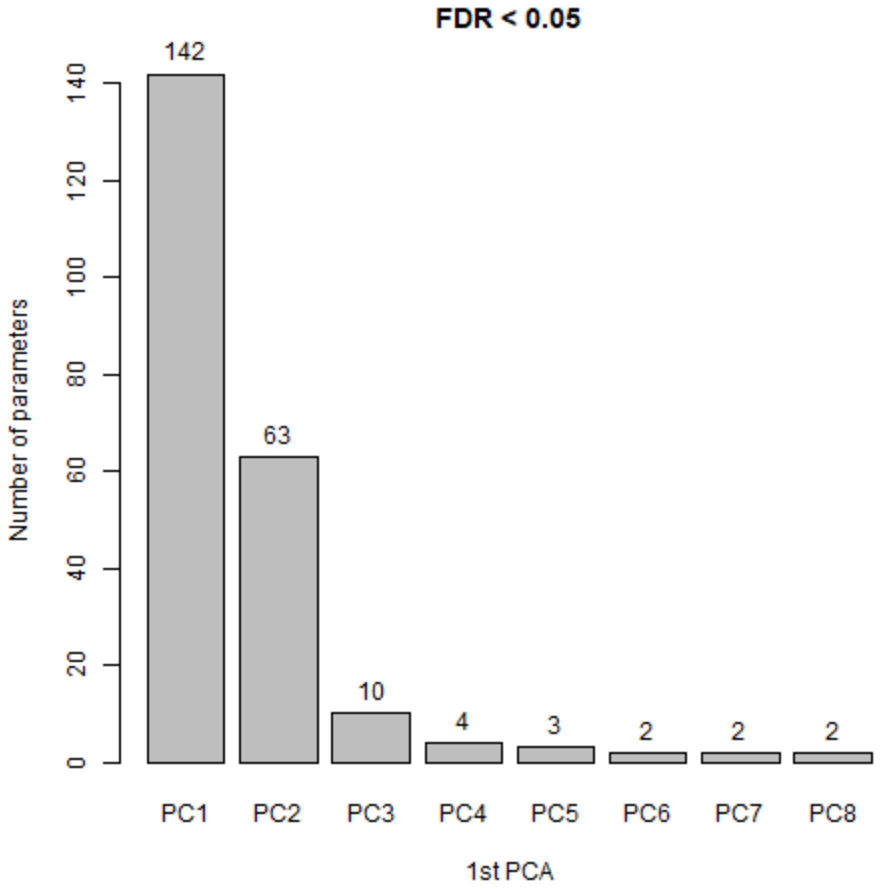

Figure S4. Number of morphological parameters significantly correlated with each principal component in the first PCA analysis (FDR = 0.05).

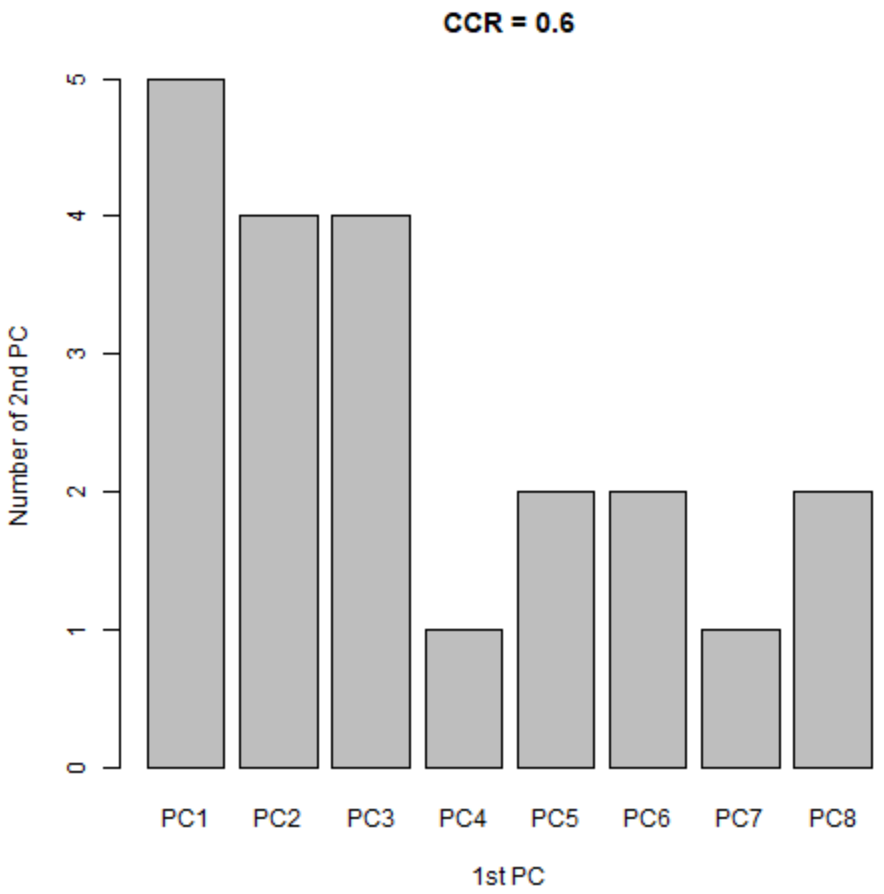

Figure S5. Number of principal components in the second PCA correlated with each principal component in the first PCA analysis (cumulative correlation ratios = 0.6).

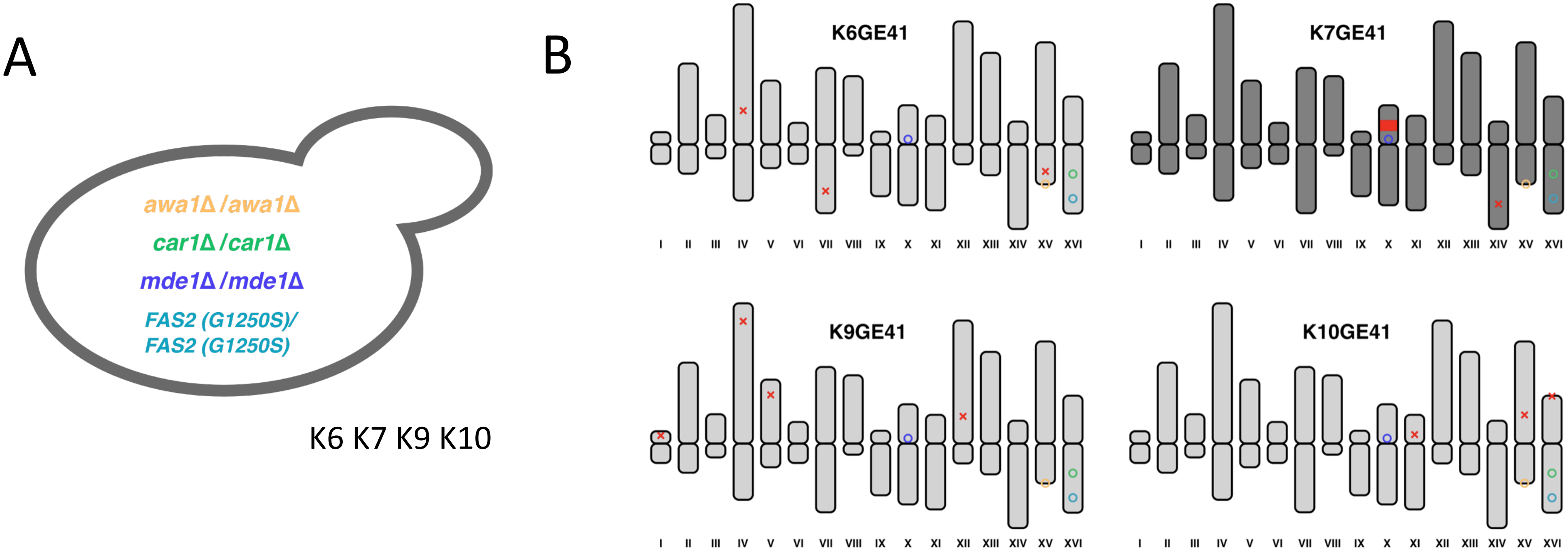

Figure S6. Genome-edited sake yeast strains used in this study. (A) Construction of genome-edited strains harboring the eight mutations, *awa1Δ/awa1Δ, car1Δ/car1Δ, mde1Δ/mde1Δ*, and *FAS2*(G1250S)/*FAS2*(G1250S). Excellent pedigrees with different genetic backgrounds (K6, K7, K9, and K10) were used as starting materials. (B) Illustration of the genetic perturbations in genome-edited sake yeast strains K6GE41, K7GE41, K9GE41, and K10GE41. Chromosomes of K7GE41 are highlighted with dark grey as it was obtained previously [12]. Yellow, green, dark blue, and skyblue circles represent *awa1Δ/awa1Δ, car1Δ/car1Δ, mde1Δ/mde1Δ,* and *FAS2*(G1250S)/*FAS2*(G1250S) mutations, respectively. Red band and red cross marks represent loss of heterozygosity and genes with unexpected mutations, respectively.

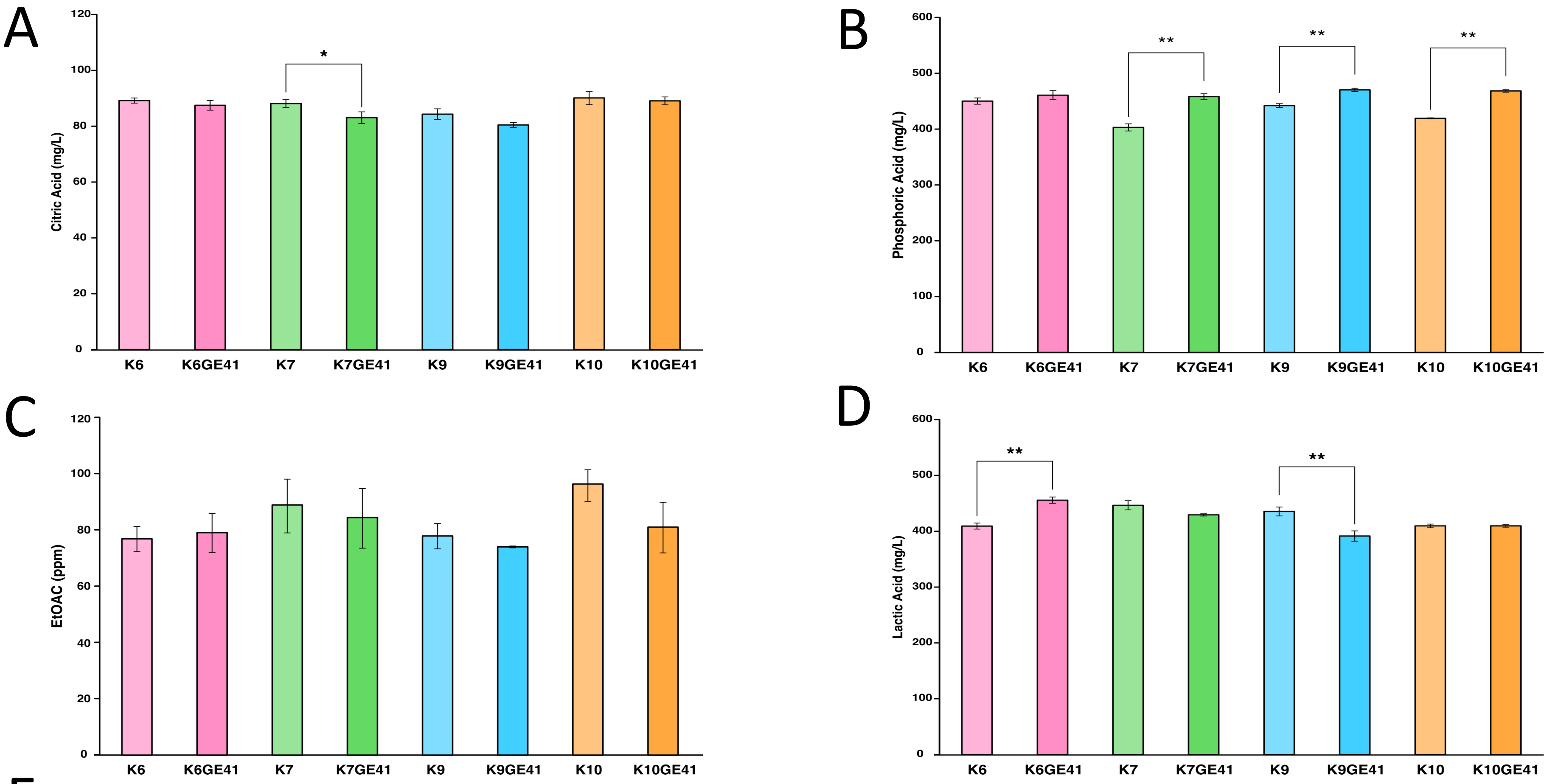

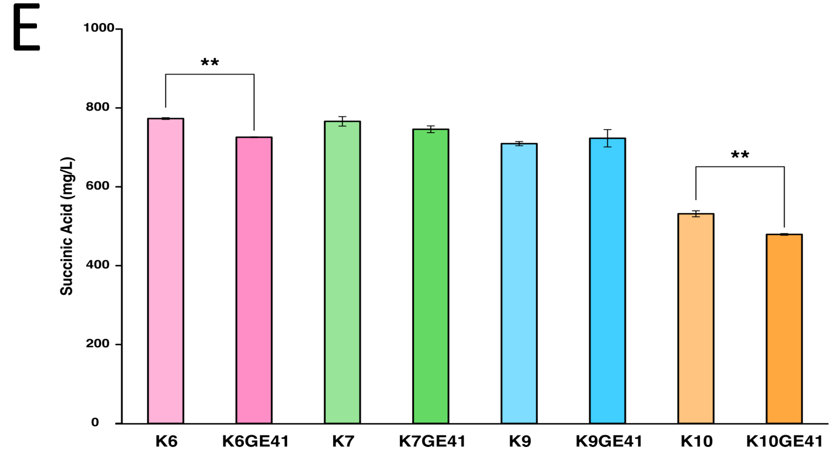

Figure S7. Measurement of citric acid (A), phosphoric acid (B), ethyl acetate (EtOAc) (C), lactic acid (D), and succinic acid (E) in sake made with genome-edited strains. Error bars indicate standard error (n = 3). Asterisks indicate Tukey's multiple comparison test (**p* < 0.05, ***p* < 0.01).

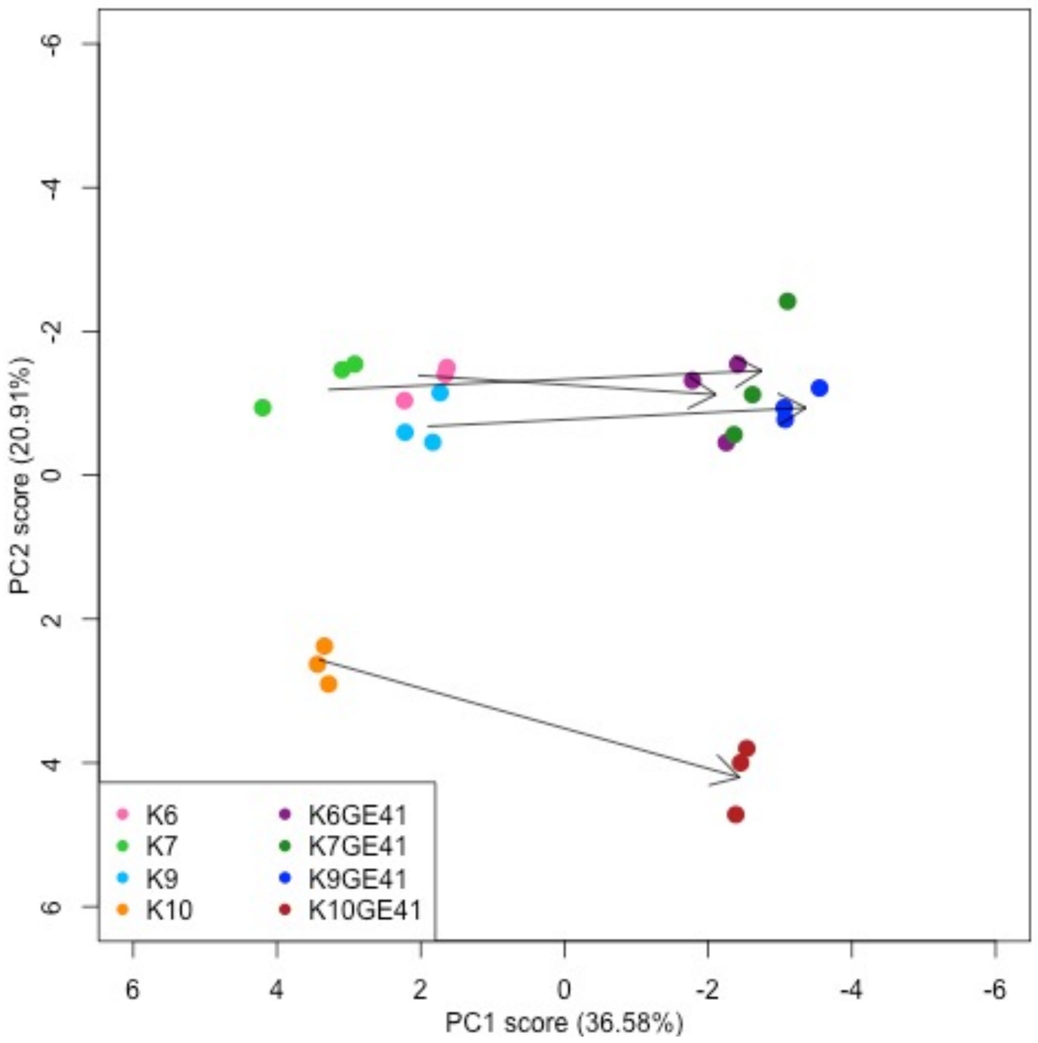

Figure S8. Principal component analysis (PCA) of sake produced by genome-edited yeast strains. Distributions of genome-edited yeast strains (K6GE41, K7GE01, K9GE41, and K10GE41) and their original pedigreed strains (K6, K7, K9, and K10) are shown in the PC1–PC2 orthogonal space after performing PCA with 21 components (PCs). Generation of genome-edited strains is indicated by arrows.

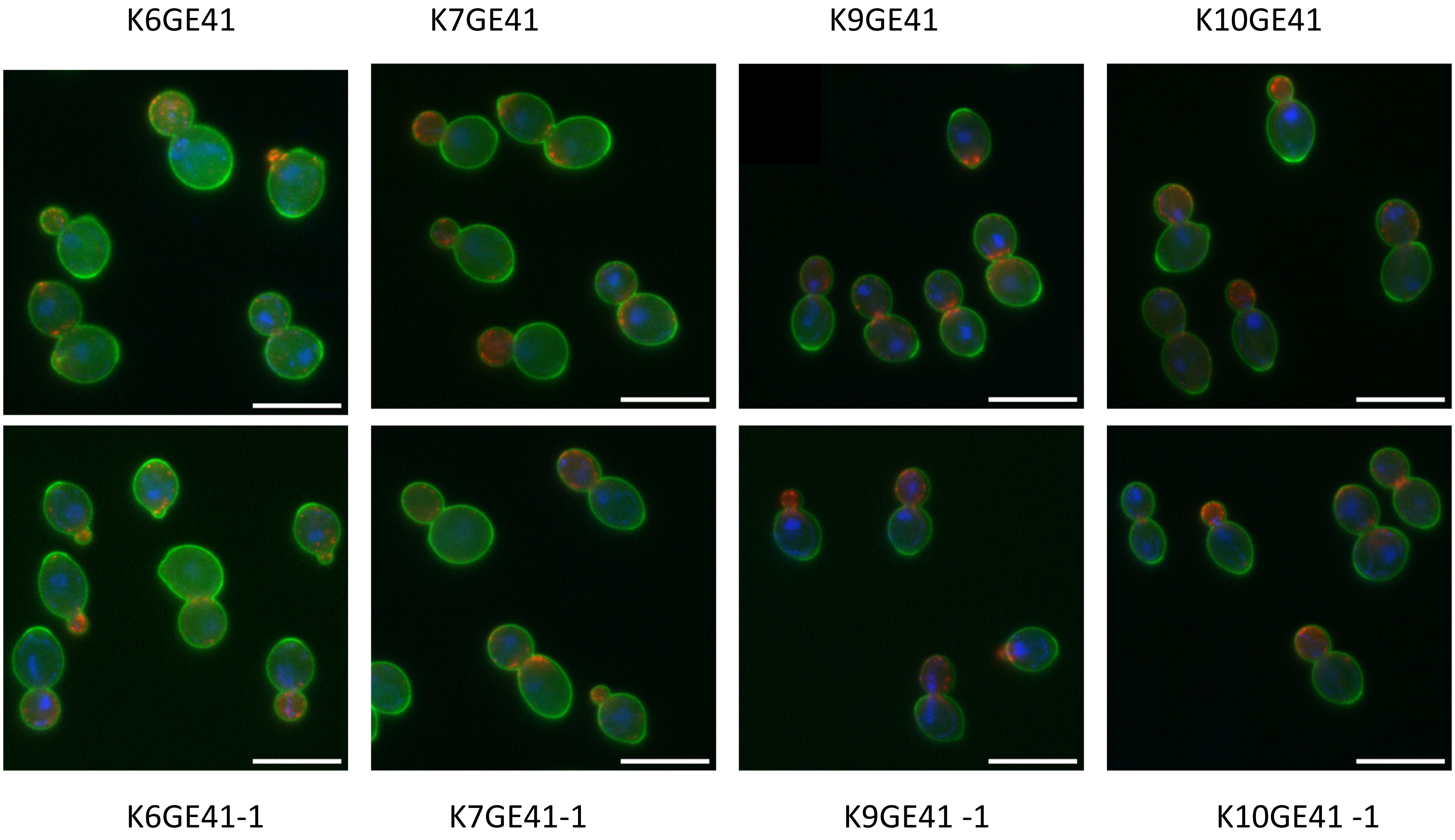

Figure S9. Morphological phenotypes of genome-edited yeast strains (K6GE41, K7GE01, K9GE41, and K10GE41) and their independently isolated genome-edited yeast strains (K6GE41-1, K7GE01-1, K9GE41-1, and K10GE41-1). The cell wall (green), nuclear DNA (blue), and actin cytoskeleton (red) were stained with FITC-ConA, DAPI, and rhodamine-phalloidin, respectively. Scale bars indicate 10 µm.

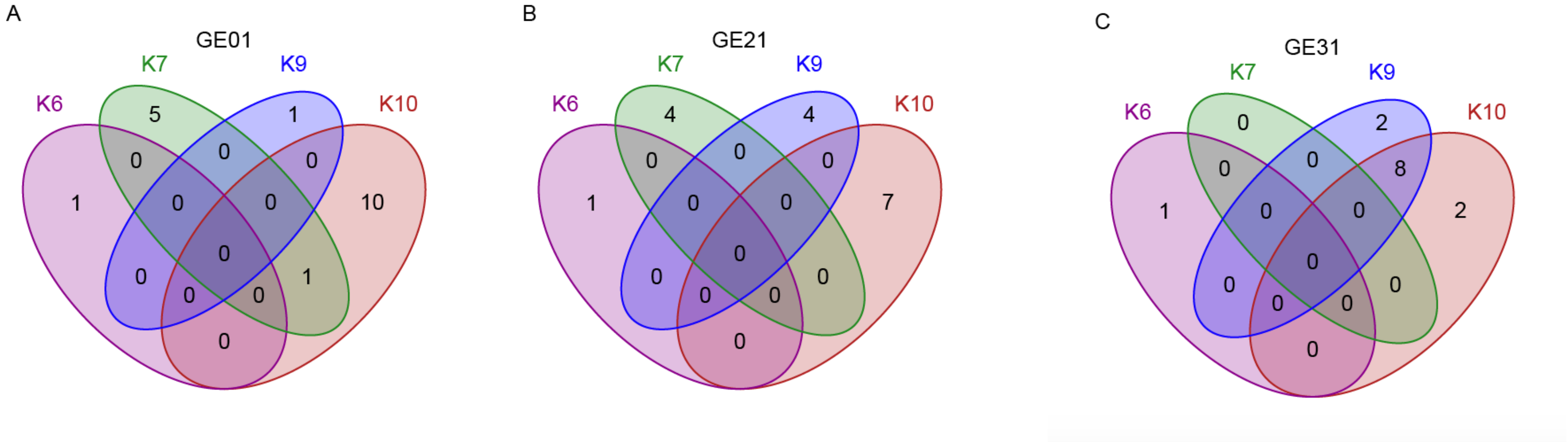

Figure S10. Venn diagram showing the number of morphological traits changed in the steps of *awa1Δ/awa1Δ* (A), *car1Δ/car1Δ* (B), and *mde1Δ/mde1Δ* (C) introduction. Wald tests were used to detect the parameters for which this step of breeding has a significant effect (FDR = 0.05). Among the 501 morphological parameters, 331 parameters were detected with this step.
